# Strategies for Hyaluronic Acid-Based Hydrogel Design in Drug Delivery

**DOI:** 10.3390/pharmaceutics11080407

**Published:** 2019-08-12

**Authors:** Sonia Trombino, Camilla Servidio, Federica Curcio, Roberta Cassano

**Affiliations:** Department of Pharmacy, Health and Nutritional Science, University of Calabria, Arcavacata, 87036 Rende, Italy

**Keywords:** hyaluronic acid, hydrogel, cancer, drug delivery, click chemistry, biomaterial

## Abstract

Hyaluronic acid (HA) is a natural, linear, endogenous polysaccharide that plays important physiological and biological roles in the human body. Nowadays, among biopolymers, HA is emerging as an appealing starting material for hydrogels design due to its biocompatibility, native biofunctionality, biodegradability, non-immunogenicity, and versatility. Since HA is not able to form gels alone, chemical modifications, covalent crosslinking, and gelling agents are always needed in order to obtain HA-based hydrogels. Therefore, in the last decade, different strategies for the design of physical and chemical HA hydrogels have been developed, such as click chemistry reactions, enzymatic and disulfide crosslinking, supramolecular assembly via inclusion complexation, and so on. HA-based hydrogels turn out to be versatile platforms, ranging from static to smart and stimuli-responsive systems, and for these reasons, they are widely investigated for biomedical applications like drug delivery, tissue engineering, regenerative medicine, cell therapy, and diagnostics. Furthermore, the overexpression of HA receptors on various tumor cells makes these platforms promising drug delivery systems for targeted cancer therapy. The aim of the present review is to highlight and discuss recent advances made in the last years on the design of chemical and physical HA-based hydrogels and their application for biomedical purposes, in particular, drug delivery. Notable attention is given to HA hydrogel-based drug delivery systems for targeted therapy of cancer and osteoarthritis.

## 1. Introduction

Hydrogels are three-dimensional, hydrated polymeric networks, formed by crosslinked hydrophilic polymers with a high affinity for water and biological fluids, capable of absorbing from 10% up to thousands of times their dry weight in water [1].

In recent years, thanks to their unique properties such as biocompatibility, biodegradability, flexibility, softness, etc., hydrogels have been widely investigated for biomedical applications like cell therapy, tissue engineering, drug delivery, and diagnostics [2]. For example, hydrogels made of pectin, carboxymethylcellulose and propylene glycol or polyethylene glycol (PEG) and propylene glycol are used as wound dressings [3,4], keratin- or polyvinyl alcohol-based hydrogels as scaffolds for cell growth [5,6], PEG-based hydrogel (DEXTENZA^®^), recently approved by the Food and Drug Administration, as ophthalmic inserts, etc. [7].

Among biopolymers, hyaluronic acid (HA) represents one of the most used in the design of hydrogels for biomedical applications due to its biocompatibility, native biofunctionality, biodegradability, non-immunogenicity, and versatility.

HA is a natural linear polysaccharide that consists of alternating units of d-glucuronic acid and *N*-acetyl-d-glucosamine, connected by β-1,3- and β-1,4-glycosidic bonds.

HA is a non-sulfated glycosaminoglycan that is widely found in the epithelial and connective tissues of vertebrates and it is the major component of the extracellular matrix (ECM) [8]. It is synthesized by hyaluronan synthase at the plasma membrane and it is then extruded to the extracellular matrix [9]. HA is found in a wide range of molecular weights from 20,000 up to several million Daltons, depending on the enzyme that catalyzes its synthesis. 

Around 30% of HA present in the body is rapidly degraded by hyaluronidases and oxidative species, while the remaining 70% is catabolized by liver and lymphatic vessels endothelial cells, with tissue half-lives going from minutes in the bloodstream to weeks in cartilage [10]. Upon physiological conditions, HA is a polyanion associated with extracellular cations (Na^+^, Ca^2+^, Mg ^2+^, K^+^) known as hyaluronan [11].

HA plays important physiological and biological roles in the human body. In the extracellular matrix of most tissues it contributes to maintain the tissue’s mechanical integrity, homeostasis, viscoelasticity and lubrication thanks to its high molecular weight and its capacity to absorb a high quantity of water [12]. Furthermore, it also plays an important role in intracellular functions; in fact, it is able to regulate, due to its binding to cell surface specific receptors (such as CD44 or RHAMM), cell adhesion, migration, proliferation, and differentiation and, consequently, processes like inflammation, wound healing, tissue development, morphogenesis, tumor progression, and metastasis [13].

For these reasons, in recent years, hydrogels built from HA have been developed and investigated for biomedical applications like tissue regeneration, tissue engineering, drug delivery, gene therapy, diagnostics, etc.

Nowadays several HA-based hydrogels are already used in medicine as dermal fillers, viscosupplements, wound dressings, etc., and the market is continuously increasing worldwide. They are now progressing in their design to be responsive to several triggers, to have various features like stability, complex structures, and biochemical cues. The aim of the present review is to highlight and discuss recent advances made in the last years on the design of chemical and physical HA-based hydrogels and their application for biomedical purposes, in particular, drug delivery.

## 2. Physical and Chemical Hydrogels 

Hydrogels can be classified into “physical” and “chemical” gels, depending on the type of bond that is formed between the polymer chains from which they derive. 

Hydrogels are called “reversible” or “physical” if the networks are formed as a result of weak physical interactions between the macromolecular chains such as ionic, H-bonding, Van der Waals interactions, hydrophobic forces, or molecular entanglements [14].

Physical hydrogels can be synthesized by warming or cooling polymer solution, mixing solution of polyanion and polycation, combining polyelectrolyte with multivalent ions of opposite charge, etc. [1]. 

Physical hydrogels are often heterogeneous, unstable, and reversible; in fact, they are not able to maintain their structural integrity and dissolve easily by changing environmental factors like temperature, pH, etc. 

Instead, hydrogels are called “permanent” or “chemical” if the polymeric chains are connected by covalent bonds [15]. For this reason, these materials, after swelling, retain their structural integrity, even if it is possible a degradation when particular bonds, sensitive to chemical or enzymatic hydrolysis, are present in the structure. 

Chemical hydrogels can be generated by crosslinking polymers with radiations, chemical crosslinkers, polyfunctional compounds, free radical generating compounds, etc. [1]. Consequently, these systems have a better chemical, mechanical, and thermal stability compared to physical hydrogels.

Even though HA, due to its conformation and molecular weight, can form molecular networks in solution, it is not able to form physical gel alone. For this reason, chemical modifications, covalent crosslinking, and gelling agents are needed in order to obtain HA hydrogels.

HA turns out to be a functional and suitable polymer for chemical modification with reactive species due to its chemical structure; in particular, the chemical modifications concern three functional groups: the carboxylic acid group, the hydroxyl group, and the amino group (after deacetylation) [16]. 

This section reviews the different modified HA macromers and chemical techniques described in the literature in the last years used for the design of physical and chemical HA-based hydrogels. 

### 2.1. Chemical Hydrogels

Chemical crosslinking turns out to be a versatile method to obtain hydrogels with excellent chemical, mechanical and thermal stability. However, this approach presents some limitations like the use of metal catalysts, photoinitiators, low reaction yield, etc. [17]. With regard to the chemical approach, HA-based hydrogels can be obtained via condensation reactions, enzymatic crosslinking, disulfide crosslinking, click chemistry, polymerization, etc. HA can be directly crosslinked by divinyl sulfone [18], glutaraldehyde [19], carbodiimide [20], bisepoxide [21], etc.; however, direct crosslinking cannot be considered suitable for hydrogels design since it requires harsh reaction condition, toxic by-products can be formed and the crosslinking agents used are cytotoxic. Nowadays, one of the most promising strategies for hydrogels preparation turns out to be click chemistry due to its high specificity, high yield, bioorthogonality, and mild reaction conditions [22].

#### 2.1.1. Diels Alder Reaction (Click Chemistry)

In recent years, a particular interest has been addressed to the Diels–Alder reaction between furan and maleimide moieties for hydrogels design due to its selectivity, efficiency, and thermoreversibility [23]. 

In this regard, Fisher et al. recently developed a HA-based hydrogel with tunable properties to use as a platform to investigate breast cancer cells invasion. The hydrogel was obtained via a Diels–Alder click reaction between furan modified HA and bismaleimide functional peptides with the aim of mimicking ECM [24]. A similar approach was reported by Yu et al. in order to obtain a 3D patterned hydrogel. In this work Diels–Alder click chemistry has been used in order to get a HA-based hydrogel that can be subsequently subjected to thiol-ene photocoupling allowing its spatiotemporal patterning [25].

#### 2.1.2. Azide-Alkyn Huisgen Cycloaddition (Click Chemistry)

The Huisgen reaction is a cycloaddition between an azide and an alkyne to produce triazoles which requires the presence of a catalyst (Cu^+^) as reported by Rostovtsev et al. [26]. In recent years it has become one of the most used strategies for hydrogels preparation thanks to its high yield, efficiency, excellent bioorthogonality, fast reaction rate, etc. [27,28]. For example, Manzi et al. fabricated nanohydrogels based on derivatives of HA and riboflavin obtained by Cu^+^ catalyzed Huisgen cycloaddition [29]. 

**Figure 1:**
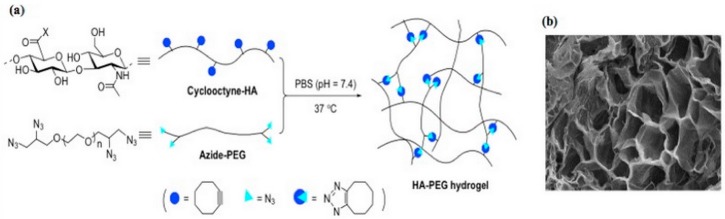
Preparation of the HA-PEG hydrogel (**a**), SEM image of the hydrogel (**b**) [31]. Reproduced with permission from Elsevier, 2017.

Despite the various advantages offered by this reaction, the use of copper as a catalyst can be problematic since it is a cytotoxic element. However, recently, it has been observed that cyclooctyne functionalized molecules are able to react rapidly with azide without the presence of copper [30]. This alternative reaction, called strain-promoted azide-alkyne cycloaddition, is currently more used for hydrogels design since it presents biosafety as well as the advantages of the previous reaction. On this matter, Fu et al. fabricated an injectable HA-PEG based hydrogel [31]. In particular, cyclooctyne modified HA was synthesized by reacting HA with 2-(aminoethoxy)cyclooctyne (Figure 1); subsequently, it was reacted with azide functionalized PEG in order to obtain the hydrogel. Interestingly, the resulting hydrogel showed fast gelation time, excellent mechanical properties, and high stability.

#### 2.1.3. Thiol-ene Photocoupling (Click Chemistry)

**Figure 2:**
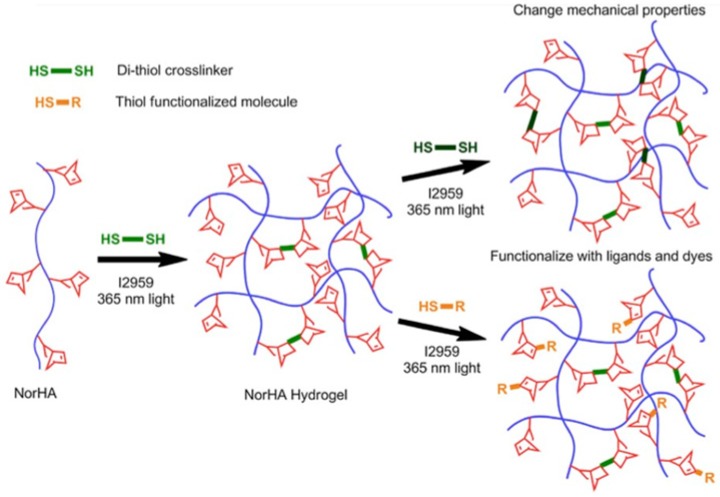
Synthesis scheme of hydrogel [37]. Reproduced with permission from Elsevier, 2013.

The thiol-ene reaction consists of a radical addition (induced by light) between a thiol and enes which has a high yield, efficiency, specificity and a fast reaction rate [32]. This method is particularly attractive for hydrogels preparation because it is solvent free and allows the hydrogels spatiotemporal control; for this reason, it is particularly investigated for the design of hydrogels to use as scaffolds for tissue engineering and cell culture or as drug delivery systems [33,34]. Different vinyl groups are employed for thiol-ene click chemistry like norbornene [35], vinyl sulfone [36], maleimide, etc. The thiol-norbornene reaction is characterized by greater specificity compared to the use of other functional groups. For example, Gramlich et al. employed this method for the synthesis of a photopatterned HA-based hydrogel by reacting norbornene modified HA (NorHA) with dithiothreitol (Figure 2) [37]. Hydrogels with an elastic modulus ranging from 1000 Pa to 70,000 Pa were obtained by varying the quantity of crosslinker. Furthermore, they reported that a secondary thiol-norbornene reaction can be performed to the hydrogel by reducing the initial amount of crosslinker.

In this context, we also find the thiol-Michael addition which, however, is characterized by lower orthogonality. In this regard, Khetan et al. designed and prepared a 3D hydrogel via a two step crosslinking process: firstly, via a thiol-Michael addition between methacrylate-maleimide functionalized HA and thiols of peptides and, subsequently, via methacrylates photopolymerization [38].

#### 2.1.4. Aldehyde-Hydrazide Coupling (Click Chemistry)

**Figure 3:**
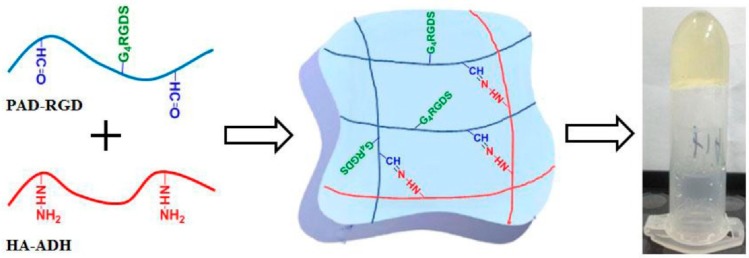
Hydrogels synthetic scheme [42]. Reproduced with permission from Elsevier, 2017.

The aldehyde-hydrazide reaction has currently attracted interest in hydrogels design due to its high efficiency, cytocompatibility, simplicity, reversibility, and mild reaction conditions [39,40,41]. In particular, covalent hydrazone crosslinked hydrogels seem to be a promising approach for tissue engineering. In this regard, Chen et al. recently developed an injectable HA-pectin based hydrogel, by reacting HA adipic dihydrazide with biofunctionalized pectin-dialdehyde, and investigated its use as a scaffold for cartilage tissue engineering (Figure 3) [42]. Interestingly, the resulting hydrogel exhibited a fast gelation rate, good mechanical properties, biocompatibility, and cytocompatibility that make it a potential platform suitable for tissue regeneration. To further improve the structural integrity of hydrazone crosslinked hydrogels, Wang et al. recently reported the design and the preparation of an elastin-like protein/HA-based hydrogel by combining two different crosslinking processes (covalent and thermal) [43]. Firstly, the hydrogel was obtained via an aldehyde-hydrazide coupling between hydrazine modified elastin-like protein and aldehyde modified HA; the use of a thermoresponsive protein allowed a secondary thermal crosslinking that improved its structural integrity and stability. The resulting hydrogel showed shear-thinning and self-healing properties, easy injectability and protection of cells from the mechanical stress of injection and for these reasons can be considered a promising candidate for stem cells delivery.

#### 2.1.5. Enzymatic Crosslinking

**Figure 4:**
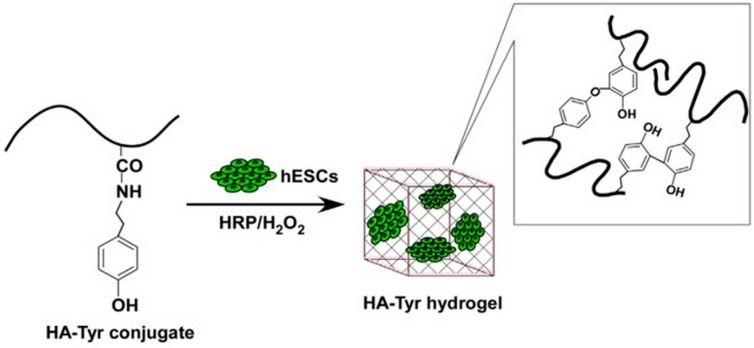
Schematic representation of hydrogels preparation and of encapsulation of human embryonic stem cells (hESCs) [48]. Reproduced with permission from Elsevier, 2015.

Enzymatic crosslinking represents an interesting approach for HA-based hydrogels preparation because it is characterized by mild reaction condition, fast gelation rate and leads to obtaining hydrogels with excellent mechanical properties [44,45]. Among different enzymes, horseradish peroxidase (HRP) turns out to be one of the most employed; it is usually employed in combination with hydrogen peroxide and the reaction can be summarized as follows: 2Ph + H_2_O_2_ → 2Ph·+ H_2_O [46]. In literature are reported various tyramine modified HA-based hydrogels which are formed as a result of an enzymatic crosslinking process that occurs through oxidation of tyramine which causes the formation of di-tyramine bonds [47]. In this regard, Xu et al. designed and fabricated HA-tyramine hydrogels by crosslinking tyramine moieties of HA in the presence of HRP and hydrogen peroxide (Figure 4) [48]. By varying the amounts of the enzyme and hydrogen peroxide, hydrogels with different mechanical strength were formed and were investigated in order to obtain a stable scaffold for stem cells culture. Interestingly, it was observed that the hydrogel with an elastic modulus of 350 Pa supported the proliferation of stem cells. A similar approach was reported by Raia et al. that enzymatically crosslinked tyramine functionalized HA and silk fibroin in order to increase the mechanical strength and the stability of tyramine-HA-based hydrogels [49]. By changing polymers concentration, hydrogels with tunable properties were obtained, resulting in versatile platforms that can be employed for various applications in tissue engineering. 

#### 2.1.6. Disulfide Crosslinking

**Figure 5:**
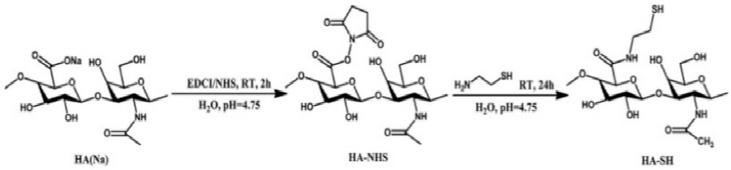
Synthesis scheme of thiol modified HA [52]. Reproduced with permission from Elsevier, 2016.

In the last years, disulfide crosslinked hydrogels have attracted growing attention because this crosslinking method presents various advantages such as biosafety, ease of execution, reversibility and permits to obtain hydrogels with *in-situ* gelation properties and responsiveness to reductant stimuli. Generally, disulfide bonds are formed via oxidation of thiols induced by air or oxidating agents like Cu(II)SO_4_ [50]. Disulfide crosslinked HA hydrogels are currently investigated for tissue regeneration thanks to their degradability since they can be cleaved enzymatically by hyaluronidase and by physiological reductants like glutathione and cysteine [51]. For example, Bian et al. proposed self-crosslinking HA-based hydrogels as scaffolds for cell culture (Figure 5) [52]. They were prepared through exposition to air of different thiolated HA derivatives which were obtained by varying the degree of thiol substitution and molecular weights of HA. Gelation rate, mechanical properties, swelling degree, and degradation were investigated and the resulting hydrogel showed excellent biocompatibility, degradation behavior and, consequently, a great potential for applications in tissue engineering. 

However, disulfide crosslinking presents some disadvantages like long reaction times, the presence of strong oxidants, etc. To overcome these limitations, an interesting strategy for disulfide crosslinked HA hydrogels preparation has been recently proposed by Velasco et al. [53]. Herein, the authors investigated the effects of the presence of various electron-withdrawing groups at the β position of thiol modified HA (cysteine, *N*-acetyl-l-cysteine). Interestingly, HA functionalized with cysteine or *N*-acetyl-l-cysteine showed fast gelation rate at physiological pH, while HA-thiol did not form any gel in the same conditions. Furthermore, the resulting hydrogels showed excellent mechanical properties and hydrolytic stability.

#### 2.1.7. Crosslinking by Radical Polymerization

**Figure 6:**
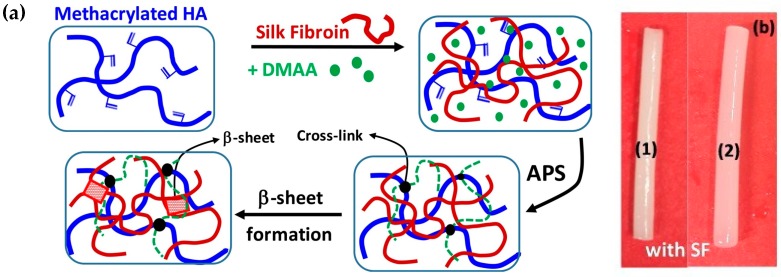
Schematic formation of HA-SF based hydrogel (**a**) and photographs of hydrogels with SF (**b**) [58]. Adapted with permission from Elsevier, 2019.

Hydrogels can be formed via radical polymerization of monomers in the presence of crosslinking agents and an initiator like a redox pair or a photoinitiator [54]. Methacrylates represent the most common groups used for HA-based hydrogels preparation and they can be introduced to HA by reacting it with glycidyl methacrylate or methacrylic anhydride [55,56]. An advantage of methacrylated HA-based hydrogels is that it is possible to tune their properties by varying HA molecular weight, the concentration of the functional monomer, the degree of substitution, etc. [57]. In this regard, Tavsanli et al. developed silk/HA-based hydrogels and investigated their mechanical properties [58]. The hydrogels were prepared by reacting methacrylated HA (MeHA) and silk fibroin (SF) in aqueous solution in the presence of *N*,*N*,*N*′,*N*′-tetramethylethylenediamine (TEMED), ammonium persulfate (APS), and *N*,*N*-dimetylacrylamide (DMMA) which has the function of connecting methacrylated HA macromers via their vinyl groups (Figure 6). Interestingly, the resulting hydrogels showed bicompatibility, excellent mechanical properties and stability thanks to the presence of SF since its β-sheet domains act as physical crosslinks.

#### 2.1.8. Crosslinking by Condensation Reactions 

**Figure 7:**
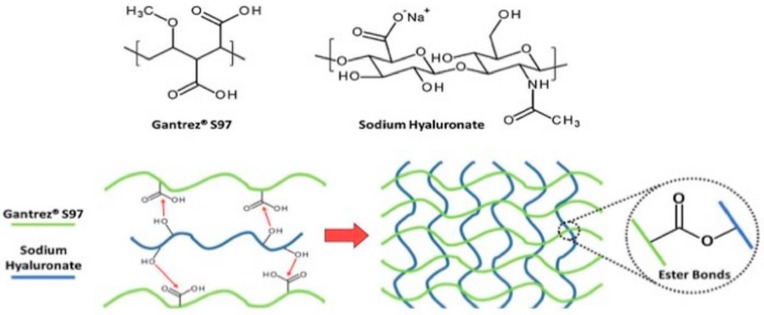
Schematic crosslinking mechanism between sodium hyaluronate and Gantrez^®^ S97 [59]. Reproduced with permission from Elsevier, 2018.

Condensation reactions are often applied for hydrogels synthesis. Considering the chemical structure of HA, among the different condensation reactions, esterification turns out to be one of the most commonly employed for hydrogels design. In this regard, Larrañeta et al. recently developed an attractive eco-friendly strategy for the synthesis of HA-based hydrogels [59]. For this purpose, Gantrex S97 was used as crosslinking agent and the hydrogels were obtained via esterification between the hydroxyl groups of HA and the carboxylic groups of Gantrex S97 (Figure 7). Since the reaction takes place in solid phase inside a microwave or an oven, the process can be considered green because it does not need organic solvents or toxic substances. Furthermore, the release capabilities and the antimicrobial properties were investigated. Interestingly, the resulting hydrogel showed a sustained release and anti-infective properties resulting in a promising candidate for the design of drug delivery systems and wound dressings.

### 2.2. Physical Hydrogels

In recent years, non-covalent bonds and supramolecular interactions have been widely investigated for hydrogels design thanks to their singular features. First of all, since these interactions are reversible, the non-covalent assembly allows to obtain hydrogels with tunable properties and responsivity to various cues like light, pH, temperature, etc. [60,61]. In contrast to covalent crosslinking, physical crosslinking leads to the formation of less mechanically and chemically stable hydrogels. However, this aspect can be considered an advantageous feature since it can be exploited in order to obtain hydrogels with shear-thinning and self-healing properties [17]. 

In this context, inclusion complexation represents one of the most used strategies for the preparation of physical gels. It can be considered the result of supramolecular interactions and structural complementarity between two molecules called “host” and “guest” [62]. Cyclodextrins are one of the most widely employed hosts which have hydrophobic cavities with a high affinity for hydrophobic guests. Several guest molecules employed in pair with cyclodextrins have been reported in literature and among these the most representative is adamantane. In this regard, Rodell et al. designed a self-assembling HA hydrogel based on supramolecular interactions between adamantane functionalized HA and β-cyclodextrin functionalized HA [63]. Hydrogel formation occurred rapidly by mixing host and guest molecules in aqueous solution. Physical properties were investigated and it has been observed that were dependent on the crosslink density and structure that can be modified by varying host and guest concentrations, molar ratio, etc. The obtained hydrogel displayed shear-thinning properties resulting in a promising injectable system. Another investigated pair for inclusion complexation broadly reported in literature is represented by α- or β-cyclodextrin and azobenzenes since *trans*-azobenzene has a high binding affinity for α- or β-cyclodextrin, while *cis*-azobenzene has a low binding affinity for them. On this subject, Rosales et al. recently developed a supramolecular HA-based hydrogel using HA functionalized with β-cyclodextrin and azobenzene [64]. It has been shown that it is possible to modulate hydrogel properties with light; in fact, upon irradiation (λ = 365 nm), isomerization of azobenzene occurs changing the binding affinity between host/guest molecules and, consequently, the network connectivity and the elastic modulus of the hydrogel. Furthermore, the release capabilities were investigated, highlighting the possibility to tune drugs release profile with light exposure. A similar approach was reported by Rowland et al. using cucurbit [8] uril and cysteine-phenylalanine as host/guest pair in order to obtain a supramolecular HA-based hydrogel [65]. Another interesting non-covalent approach for HA-based hydrogels design is represented by functionalization of HA with hydrophobic molecules in order to render it amphiphilic and consequently determine the macromers self assembly in nanogels. In this regard, Montanari et al. designed HA-based hydrogels via self-assembly of macromers in water after the functionalization of HA with cholesterol [66]. In particular, the self-assembly of macromers occurs due to the hydrophobic interactions between the cholesterol cores and the hydrophilic interactions between the shells formed by HA.

Moreover, the use of gelling agents in combination with HA can be considered a valid strategy for HA physical hydrogels design. For example, Jung et al. recently reported the preparation of a thermosensitive hydrogel based on HA and Pluronic F-127 [67]. Pluronic F-127 is a triblock copolymer able to form rapidly thermoresponsive hydrogels which, however, are unstable in physiological conditions due to their low mechanical strength. To overcome this problem, in this study HA was mixed with Pluronic F-127 in water in order to obtain a hydrogel with improved structural integrity and stability due to the hydrophobic interactions that occur between acetyl groups of HA and methyl groups of Pluronic F-127. Interestingly, the resulting hydrogel not only showed an increased mechanical strength but also a sustained drug release, reducing the typical burst release observed in Pluronic F-127-based hydrogels.

## 3. HA-Based Hydrogels for Biomedical Applications

Currently, HA-based hydrogels are widely investigated for biomedical purposes like drug delivery, tissue engineering, regenerative medicine thanks to their biocompatibility, biodegradability, non–immunogenicity, responsivity to various cues, and tunable properties [68,69,70]. This section reviews the main biomedical applications of HA-based hydrogels reported in literature in the last few years, focusing in particular on drug delivery.

### 3.1. Drug Delivery

HA-based hydrogels are particularly interesting for drug delivery since, in addition to above cited features, allow to have a controlled and targeted drug release in response to different triggers that turns out to be attractive when aiming for targeted therapy [71,72].

#### 3.1.1. Stimuli-Responsive Hydrogels

Various smart hydrogels responsive to different environmental factors such as pH, temperature, light, biochemical molecules have been developed as drug delivery systems. With regard to stimuli-responsive platforms, physical crosslinking is preferred since supramolecular interactions are reversible and consequently results easier to tune hydrogels properties.

In this context, Highley et al. recently proposed an interesting strategy for the design of a near infrared light (NIR)/temperature responsive platform [73]. In this study, the platform was prepared in a microfluidic mixing device by combining gold nanorods with a HA supramolecular hydrogel obtained via the inclusion complexation of β-cyclodextrin and adamantane. The presence of nanorods caused heating in response to NIR irradiation causing consequently the breakage of supramolecular interactions and the networks disruption. The release capability of the resulting platform in response to different NIR exposures was evaluated, and interestingly it has been observed that the drug release can be modulated by varying two parameters: irradiation time, and light intensity; specifically, the quantity of the molecule released from the platform increased with increasing power and irradiation time.

Inclusion complexation employing azobenzene/cyclodextrin as host/guest pair has been broadly investigated for photoresponsive hydrogels design since the supramolecular host/guest interactions can be disrupted upon *trans-cis* photoisomerization of azobenzene induced by ultraviolet (UV) light [74]. An interesting example of a photoresponsive drug delivery platform has been described by Rosales et al. [64]. Thus, HA was functionalized with azobenzene and β-cyclodextrin in order to obtain self-assembled hydrogels. The resulting hydrogels showed reversible changes in crosslink density and, consequently, in mesh size, upon UV exposures. These changes were exploited to modulate drug release profiles and, therefore, the release capability upon different UV irradiations was evaluated. In particular, a fluorescently labeled protein was loaded as a model drug and it has been observed that upon irradiation hydrogels released a double amount of protein compared to the non irradiated ones.

Even if physical crosslinking is preferred for smart hydrogels preparation, also the chemical approach has been recently investigated. In this regard, Kwon et al. reported the preparation of pH-sensitive hydrogels based on hydroxyethyl cellulose and hyaluronic acid and investigated their potential use as transdermal delivery systems for the treatment of skin lesions [75]. In this study, the hydrogels were synthesized via Michael addition between HA and hydroxyethyl cellulose by using divinyl sulfone as crosslinking agent and their physicochemical properties were investigated. Hydrogels were then loaded with isoliquiritigenin, which has antimicrobial activity, and its release efficiency has been investigated by in vitro measurements at different values of pH. Experimental data showed that the quantity of isoliquiritigenin released increased with increasing pH beyond 7 due to electrostatic repulsions between the carboxylate groups of HA that cause consequently an increase of mesh size.

#### 3.1.2. HA-Based Hydrogels for Targeted Cancer Treatment

As already reported, HA regulates different cellular functions such as cell adhesion, differentiation, migration, proliferation, etc., which arise as a result of HA binding to specific membrane surface receptors, called hyaldherins, like CD44, LYVE-1, and RHAMM [76]. In particular, CD44 is the main receptor involved in cellular proliferation, differentiation, and migration pathways and consequently in tumor progression and metastasis; moreover, it is overexpressed in various types of tumors like melanoma, chondrosarcoma, breast, gastrointestinal, prostate, bladder, lung, and pancreatic cancers, and different studies have reported a relationship between CD44 expression and poor prognosis [77]. For these reasons, HA has recently emerged as a promising molecule for the design of anticancer drug delivery systems for active targeting of malignant tumors [78]. Comprehensive reviews by Huang et al. and Choi et al. supply an interesting description of HA-based drug delivery systems developed for targeted cancer treatment [79,80].

In the last years, several HA-based hydrogels have been studied for the delivery of different antitumor drugs such as doxorubicin, paclitaxel, cisplatin, etc., in order to improve their antitumor activity and reduce their systemic side effects [81,82]; some of the most interesting and recent examples will now be presented.

**Figure 8:**
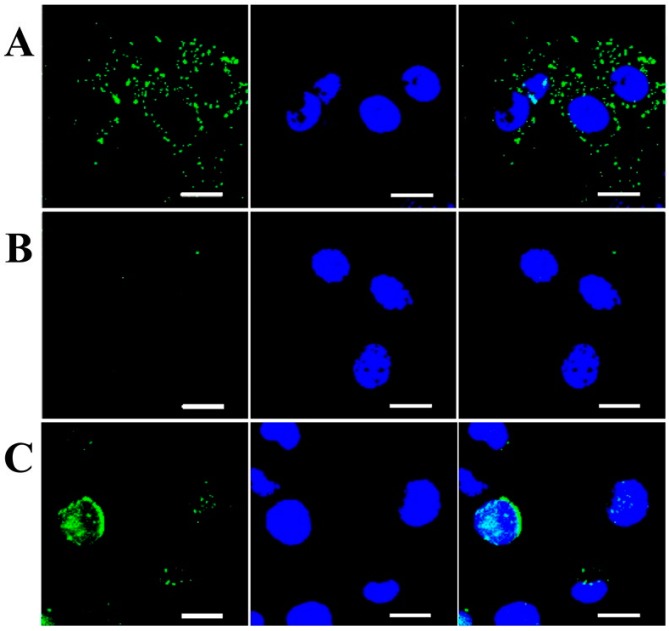
Confocal laser scanning microscopy of (**a**) A549, (**b**) NIHT3T, and (**c**) H22 cells incubated with FITC-labeled HA nanogels [83], The scale bar =10μm. Reproduced with permission from Elsevier, 2015.

Aiming at preparing a suitable drug delivery platform for the targeted release of doxorubicin, Yang et al. prepared various HA-based nanogels via copolymerization of methacrylated HA with di(ethylene glycol) diacrylate [83]. Nanogels with a diameter of about 70 nm and a spherical shape were obtained, which were then loaded with doxorubicin by an incubation method. In vitro studies showed a CD44-dependent cellular uptake and consequently a greater internalization of nanogels in tumor cell lines that overexpress CD44 receptor (Figure 8).

Furthermore, the nanogels showed higher accumulation in the tumor site and a superior antitumor activity compared with the free doxorubicin, resulting in a promising drug delivery system for cancer therapy.

A noteworthy further example of doxorubicin delivery platform has been reported by Jhan et al. [84]. In this study, injectable thermosensitive hydrogels based on Pluronic F-127 and HA-doxorubicin nanocomplexes were prepared via physical mixing and their physiochemical properties were investigated. The doxorubicin release profile was studied in vitro, while nanogels antitumor activity both in vivo and in vitro. Experimental data showed a pH sensitive and sustained release of doxorubicin from hydrogels with a faster release rate at tumoral pH. Moreover, hydrogels showed an excellent cytotoxic activity against tumor cell lines that overexpress CD44 receptor and a high affinity targeting to the lymphonodes, resulting in promising injectable formulations for the treatment of local and metastatic tumors.

Furthermore, in this context, an efficient approach for metastatic breast cancer treatment has been recently described by Chen et al. [85]. In particular, the authors prepared saporin loaded epidermal growth factor receptor (EGFR) and CD44 dual targeted HA nanogels by combining inverse nanoprecipitation and tetrazole-alkene photocoupling. Nanogels with a diameter of about 160 nm and a spherical shape were obtained and evaluated for the treatment of metastatic breast cancer. In vitro studies showed an excellent internalization of nanogels in 4T1 breast cancer cell line that overexpresses both EGFR and CD44. Furthermore, in vivo studies in metastatic 4T1-luc breast tumor bearing mice displayed that the nanogels enhanced the therapeutic efficacy of saporin, showing an excellent inhibition of tumor growth and lung metastasis.

Currently intraperitoneal (IP) chemotherapy is emerging as an efficient strategy for the treatment of solid tumors present in the peritoneal cavity [86]. However, the delivery systems suitable for the IP chemotherapy should have some requirements like biocompatibility, biodegradability, non-immunogenicity and the capacity to control the drug release. Since HA hydrogels have these features, they are now also studied for the design of drug delivery platforms for IP chemotherapy. In this regard, Cho et al. recently reported the design of in situ crosslinkable HA-based gels and evaluated their potential use as IP carriers of platinum for the treatment of ovarian cancer [87]. Firstly, they prepared platinum loaded nanoparticles that were incorporated in HA-based hydrogels obtained via the aldehyde-hydrazide coupling. The obtained platforms showed sustained platinum release, good anti-tumor activity and a long permanence in the peritoneal cavity resulting attractive for IP chemotherapy of ovarian cancer.

Unfortunately, one problem that can occur sometimes when using hydrogels as drug delivery systems is the burst release [88]; with the attempt to reduce the burst release of drugs from hydrogels, Zhang et al. proposed an innovative strategy for HA-based hydrogels design [89]. In this regard, they prepared multilayer hydrogel capsules based on chitosan, HA and doxorubicin via the ionotropic crosslinking method. Release studies concerning these hydrogels showed a pH-sensitive, controlled release of doxorobucin with a notable reduction of the burst release due to their particular structure; in fact, the multilayer structure reduced the drug concentration gradient between the capsules external layer and the surrounding environment limiting the release of doxorubicin adsorbed on the surface of the hydrogels.

#### 3.1.3. HA-Based Hydrogels for Osteoarthritis Treatment

Osteoarthritis is an inflammatory, chronic joint disorder characterized by progressive cartilage erosion. Currently, osteoarthritis treatment is limited to anti-inflammatory drugs administration, viscosupplementation and, if necessary, prosthesis graft at the last stage [90].

As already reported, HA performs multiple biological functions: at the articular level, thanks to its viscoelasticity, it acts as a lubricant, increasing the viscosity of the synovial fluid, and as a cushioning, allowing the separation of the articular surfaces under load.

HA presents also chondroprotective and anti-inflammatory effects [91]. Furthermore, some studies reported the overexpression of CD44 receptor on human articular chondrocytes [92]. For these reasons, HA-based hydrogels are widely investigated and used as viscosupplements and delivery platforms for osteoarthritis treatment.

**Figure 9:**
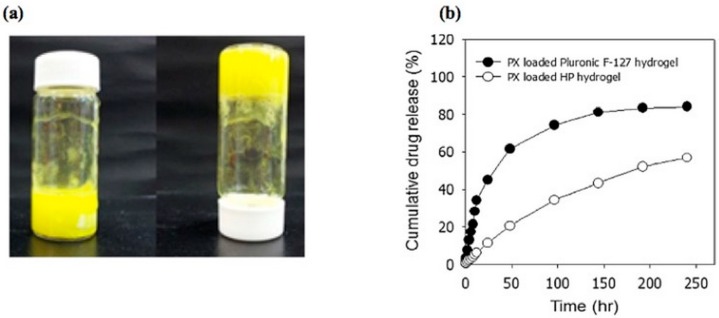
Photographs of the hydrogel (**a**) and in vitro Paclitaxel release from Pluronic F-127 and Pluronic F-127/HA-based hydrogels (**b**) [67]. Reproduced with permission from Elsevier, 2016.

In this regard, Jung et al. recently reported the preparation of an injectable thermosensitive hydrogel via physical mixing of HA and Pluronic F-127 [67]. As previously reported, Pluronic F-127 is a gelling agent able to rapidly form thermoresponsive hydrogels that however are instable in physiological conditions due to their low mechanical strength. To overcome this problem, in this study, HA was physically mixed with Pluronic F-127 in order to obtain a hydrogel with improved structural integrity and stability due to the hydrophobic interactions that occur between HA and Pluronic. The hydrogel was then loaded with piroxicam and evaluated for the treatment of osteoarthritis. In Vitro release studies showed a 10 days sustained and slow release of piroxicam from HA-Pluronic F-127 hydrogel, while Pluronic F-127 based hydrogels displayed a faster release behavior (Figure 9). Furthermore, in vivo pharmacokinetic studies reported an increase of drug bioavailability and half-life in comparison to a commercial Paclitaxel formulation.

In addition to the classical therapeutic approaches, nowadays gene silencing of signal molecules, proteins, enzymes, that play an important role in osteoarthritis degenerative events, is emerging as a promising strategy for osteoarthritis treatment.

In this regard, Cai et al. recently developed a HA-based hydrogel loaded with gapmer antisense oligonucleotides that was studied for silencing target gene expression in osteoarthritis [93]. In particular, the hydrogel was prepared via Schiff reaction between aldehyde modified HA and chitosan, and subsequently, it was loaded with a gapmer oligonucleotide, obtaining a high encapsulation efficiency (~97%), in order to reduce COX-2 expression. In vitro studies showed a sustained release of COX-2 gapmer up to five days with a low burst release and, furthermore, a 10–14 days long COX-2 gene silencing activity in osteoarthritis chondrocytes. A similar approach was recently reported by Garcia et al. in order to obtain a HA-based hydrogel for the delivery of chondrocytes and antisense oligonucleotides [94]. In this case hydrogels made of HA and fibrin were loaded with an antisense oligonucleotide in order to modulate the expression of genes that codify for ADAMTS (A Disintegrin and Metallo Proteinase with Thrombospondin Motifs) enzymes which showed to play a key role in causing proteoglycans loss in osteoarthritis [95]; chondrocytes were also incorporated in the scaffolds in order to have both regenerative and therapeutic effects. In Vitro studies showed 14-day long sustained release and an efficient and long genes inhibition in both incorporated and resident chondrocytes. These features make this platform a potential formulation for osteoarthritis treatment.

## 4. Conclusions

In recent decades, HA has emerged as an attractive molecule for hydrogels design thanks to its biocompatibility, native biofunctionality, biodegradability, non-immunogenicity, and versatility. Nowadays, several HA-based hydrogels are already used in medicine as dermal fillers, viscosupplements, wound dressings, etc., and the market is continuously increasing worldwide.

Since HA is not able to form gels alone, new crosslinking methods, like click chemistry reactions, disulfide crosslinking, enzymatic crosslinking, or supramolecular assembly methods like inclusion complexation, functionalization with lipophilic molecules, etc., have been widely investigated resulting in efficient strategies for chemical and physical hydrogels design. However, these current synthetic strategies present several advantages as well as some limitations. For example, various chemical crosslinking techniques often require organic solvents, metal catalysts, reaction by-product can be formed, etc. Thus, in this respect, research is aimed at developing new advantageous synthetic strategies.

HA-based hydrogels turn out to be versatile platforms ranging from passive and static matrices to smart, stimuli-responsive platforms with tunable properties and consequently they showed to have great potential as drug delivery systems, scaffolds for tissue engineering and regenerative medicine, and so on. In particular, their tunability is exploited for the design of platforms for controlled and targeted drug delivery; in this regard, a notable attention is given to inclusion complexation employing cyclodextrin and azobenzene as a host/guest pair, which allows obtaining phototoresponsive drug delivery systems. Furthermore, since HA receptors were found to be overexpressed on various tumor cells and on chondrocytes, different HA-based hydrogels have been broadly developed for these purposes, showing promising results for targeted cancer and osteoarthritis therapies.

Nowadays, one of the main goals is the design of intelligent hydrogels with various features like stability, complex structures, biochemical cues and responsive to several triggers that, surely, will find a place into clinical practice.
